# Extreme hyperferritinemia without associated HLH in a patient with T‐cell lymphoma

**DOI:** 10.1002/ccr3.6562

**Published:** 2022-11-16

**Authors:** Grace Lau, Sean C. Dougherty, Lisa Friedman, Brian Wispelwey

**Affiliations:** ^1^ Department of Internal Medicine University of Virginia Charlottesville Virginia USA; ^2^ Department of Pathology University of Virginia Charlottesville Virginia USA

**Keywords:** acute liver injury, hemophagocytic lymphohistiocytosis, hyperferritinemia, iron overload, T‐cell lymphoma

## Abstract

Extreme hyperferritinemia has historically been associated with a short list of rare diagnoses, including hemophagocytic lymphohistiocytosis (HLH). However, hyperferritinemia is not specific for HLH in the adult population. Among other more common causes, T‐cell lymphoma and other malignancies warrant evaluation prior to considering more rare diagnoses.

## 1 INTRODUCTION

Ferritin is a predominantly cytosolic protein intricately involved in the regulation of the body's iron stores.^1^ In the absence of concomitant inflammation, ferritin has been shown to be both sensitive and specific for the diagnosis of iron deficiency.^2^ As such, it is a commonly obtained laboratory test in primary care and hospitalized patients and is often abnormal. Established reference ranges for ferritin vary based on the assay being utilized and the patient's sex, though the upper limit of normal is generally 200 ng/ml in men and 300 ng/ml in women.^3^

Marked elevations in serum ferritin levels (10,000–50,000 ng/ml) are relatively specific for hemophagocytic lymphohistiocytosis (HLH) in the pediatric population and have historically been thought to be similarly exclusive to inflammatory conditions such as adult‐onset Still's disease, HLH, and macrophage activation syndrome (MAS) in adults.^4^, ^5^, ^6^, ^7^ However, other conditions causing hyperferritinemia, such as iron overload, renal failure, hepatocellular injury, infection, and malignancy, are far more common in this population. Several studies have shown that hyperferritinemia is not specific for HLH in adults; hematologic malignancies, particularly T‐cell malignancies, represent a significant number of cases of markedly elevated ferritin levels.^8^, ^9^, ^10^, ^11^, ^12^ In the existing literature, there are several case reports of marked hyperferritinemia in patients diagnosed with T‐cell lymphoma. However, reported cases have all been found to have associated secondary HLH.^13^, ^14^, ^15^, ^16^, ^17^, ^18^, ^19^, ^20^

In this clinical case report, we detail the presentation, evaluation, and diagnosis of a case of hyperferritinemia without associated HLH in a patient with T‐cell lymphoma, presumed anaplastic large cell type, highlighting the broad differential diagnosis associated with a significantly elevated ferritin level. To our knowledge, this is one of few cases of T‐cell lymphoma with associated hyperferritinemia to lack concomitant HLH. We believe this case highlights the possibility of a spectrum of disease between marked hyperferritinemia associated with hematologic malignancy and hematologic malignancy‐associated HLH, as well as the need for prompt recognition of hematologic malignancy in order to avoid delay in diagnosis and possible progression to more advanced and/or aggressive disease.

## 2 CASE PRESENTATION

A 68‐year‐old female patient with a past medical history of monoclonal gammopathy of undetermined significance (MGUS), essential hypertension, unspecified mood disorder, and chronic kidney disease stage IV presented in the setting of encephalopathy of 3 weeks duration. Per the patient's family members, the patient had an acute change in mental status 3 weeks ago that had since progressively worsened into somnolence, difficulty speaking, and generalized weakness. She did not have fever, chills, dysuria, neck pain, nausea, vomiting, or diarrhea. Prior to presenting to our tertiary care center, she had been evaluated at two other hospitals and was diagnosed with an acute kidney injury resulting in medication‐related encephalopathy before being discharged home. Despite subsequent changes to a number of psychiatric medications she was prescribed, her altered mental status failed to improve.

The patient had been functional and independent with all activities of daily living preceding the change in mental status noted by her family. In regard to her past medical history, she had been diagnosed with MGUS (IgG L spike) at age 65 and followed with hematology since that time without need for clinical intervention. Her psychiatric history was notable for reported bipolar disorder and schizophrenia; however, the exact diagnoses remained unclear. The patient was prescribed oxcarbazepine and olanzapine as an outpatient, both of which had been titrated down and modified over the preceding several weeks due to ongoing encephalopathy and concern for medication side effect.

On physical examination, her temperature was 36.6 degrees celsius, heart rate was 99 beats per minute, blood pressure was 141/69 mm Hg, respiratory rate was 18 breaths per minute, and oxygen saturation was 99% on room air. She was only oriented to person, but not place or time, and unable to follow commands. She did not have any focal neurologic deficits, palpable hepatosplenomegaly, or lymphadenopathy. She was only intermittently able to converse throughout the encounter.

A complete blood count revealed a white blood cell count (WBC) of 6.06 × 10^9^/L, hemoglobin of 6.7 g/dl (baseline 8 g/dl) with a mean corpuscular volume of 82.5, and platelet count of 213 × 10^9^/L. A complete metabolic panel revealed a sodium of 131 mEq/L, calcium of 11.3 mg/dl, creatinine of 2.2 (baseline 1.9–2.2), aspartate aminotransferase (AST) of 72 U/L, and alkaline phosphatase (ALP) of 557 U/L. Iron studies obtained for evaluation of the patient's anemia, and prior to any blood transfusions, revealed an iron of 56 ug/dl and markedly elevated ferritin (>40,000 ng/ml). See Table 1 for complete laboratory results.

**TABLE 1:** Complete laboratory analysis

Test	Result	Reference Range
Complete Blood Count		
White blood cells (x10^9^/L)	6.06	4000‐11,000
Hemoglobin (g/dl)	6.7	12.0–16.0
Hematocrit (%)	20.4	35.0–47.0
Platelets (x10^3^/L)	213	150–450
Complete Metabolic Panel		
Sodium (mmol/L)	131	136–145
Potassium	4.9	3.4–4.8
Chloride	94	98–107
Bicarbonate	20	23–31
Blood urea nitrogen (mg/dl)	74	10–20
Creatinine	2.2	0.6–1.1
Calcium	11.3	8.5–10.5
Alkaline phosphatase (U/L)	557	40–150
Gamma Glutamyltransferase (U/L)	571	<38
AST (U/L)	72	<35 U/L
ALT (U/L)	39	<55 U/L
Total bilirubin (mg/dl)	0.3	0.3–1.2
Cerebrospinal fluid		
Color	Colorless	Colorless
WBC (U/L)	0	0–5
RBC Tube #1 (U/L)	44	0–5
Protein (mg/dl)	38	15–25
Glucose (mg/dl)	101	40–70
Lactate (mmol/L)	1.7	0.5–2.2
Bacterial culture	No growth	No growth
Varicella Zoster Virus PCR	Negative	Negative
Miscellaneous		
Hepatitis B surface antigen	Negative	Negative
Hepatitis B core total antibody	Negative	Negative
Hepatitis C antibody	Negative	Negative
HIV	Negative	Negative
Anti‐Smooth Muscle antibody	Negative	Negative
Anti‐Mitochondrial antibody	Negative	Negative
Antinuclear antibody	Negative	Negative
Iron (ug/dl)	56	40–145
Percent Saturation	40	16–48
Ferritin (ng/ml)	>40,000	5–200
Haptoglobin (mg/dl)	390	30–200
Lactate dehydrogenase (U/L)	2224	125–250
Ammonia (micromol/L)	33	11–32
Oxcarbazepine level (mcg/ml)	47	10–35
TSH (mIU/L)	0.75	0.45–4.50
Human T‐lymphotropic virus I/II antibody	Negative	Negative

A lumbar puncture was performed showing normal glucose as compared to serum glucose (101, normal range 40–70 mg/dl), absence of WBC, normal protein (38 mg/dl), mild red blood cells (RBC) (44 tube 1 > 14 tube 2), negative varicella zoster virus and herpes simplex virus PCR, and negative bacterial cultures. An oxcarbazepine level was obtained on admission and found to be elevated (47 mcg/ml, normal range 10–35 mcg/ml). A peripheral blood smear revealed a normal WBC number and morphology, no blasts, no immature forms, normal size/shape of RBC, and normal size and number of platelets. Given acute worsening of her chronic anemia and marked elevations in serum ferritin, evaluation for HLH was conducted; this revealed normal triglycerides (154 mg/dl), high fibrinogen (967 mg/dl), elevated erythrocyte sedimentation rate (77 mm/h, normal range 0–30 mm/h) and C‐reactive protein (34.7 mg/dl, normal range <0.5 mg/dl), and negative human immunodeficiency virus.

Imaging evaluation was notable for a computed tomography (CT) head showing chronic microvascular ischemic disease, a CT chest/abdomen/pelvis that was negative for infection, malignancy, or splenomegaly, and magnetic resonance imaging (MRI) of the brain with acute and chronic infarcts. Neurology was subsequently consulted, and the location and pattern of acute infarcts were thought to unlikely be the only cause of her encephalopathy. An electroencephalogram was completed and negative for seizures or seizure‐like activity.

Given low concern for HLH and absence of notable malignancy on laboratory/imaging evaluation, further investigation of hepatic dysfunction as a potential cause of hyperferritinemia was performed, as the patient's AST and ALP continued to rise over several days in the hospital. Evaluation was notable for a negative viral hepatitis panel, negative anti‐smooth muscle and antimitochondrial antibodies, and MRI of the abdomen with evidence of secondary hemochromatosis in the liver and spleen. There was notable diffuse signal abnormality throughout the lumbar spine that was concerning for metastatic disease or an infiltrative process also observed on the MRI.

## 3 DIFFERENTIAL DIAGNOSIS, INVESTIGATIONS, AND TREATMENT

The differential diagnosis for extremely elevated serum ferritin levels in the adult population is broad and can generally be broken down into three major categories: inflammatory conditions, total body iron storage overload, and hepatocellular disease resulting in release of iron via cellular injury.^8^, ^9^, ^10^, ^11^, ^12^ Despite a historical association between hyperferritinemia and HLH, only a minority of cases of extremely elevated ferritin levels are the result of HLH, as this is a clinically rare entity.^8^, ^10^, ^12^

Inflammatory syndromes resulting in hyperferritinemia include underlying bacterial, viral, or fungal infection, autoimmmune disease such as systemic lupus erythematosus or adult‐onset Still's disease, malignancy (particularly hematologic malignancies), HLH, and MAS. Our patient did not have any identifiable underlying infection, as evidenced by negative blood, urine, and cerebrospinal fluid cultures. An anti‐nuclear antibody was obtained and negative, aruging against systemic lupus erythematosus. As noted above, HLH was considered and evaluated extensively; however, the patient only had one out of eight diagnostic criteria for HLH (ferritin >500 ug/L).^5^ Given the diffuse signal abnormality observed in the lumber spine on MRI that was concerning for malignancy or an infiltrative process, malignancy was the highest on the differential diagnosis.

Processes resulting in total body iron overload were also considered as a cause of hyperferritinemia in this patient; however, she had not received frequent blood transfusions and did not have a family history of hemochromatosis. Genetic testing for hemochromatosis was therefore not obtained. In regard to hepatocellular disease, she did not have a history of significant alcohol use disorder, and hepatitis B and C serologies were negative for acute infection. Evaluation for autoimmune hepatitis was also unremarkable, as anti‐smooth muscle and antimitochondrial antibodies were negative.

**FIGURE 1:**
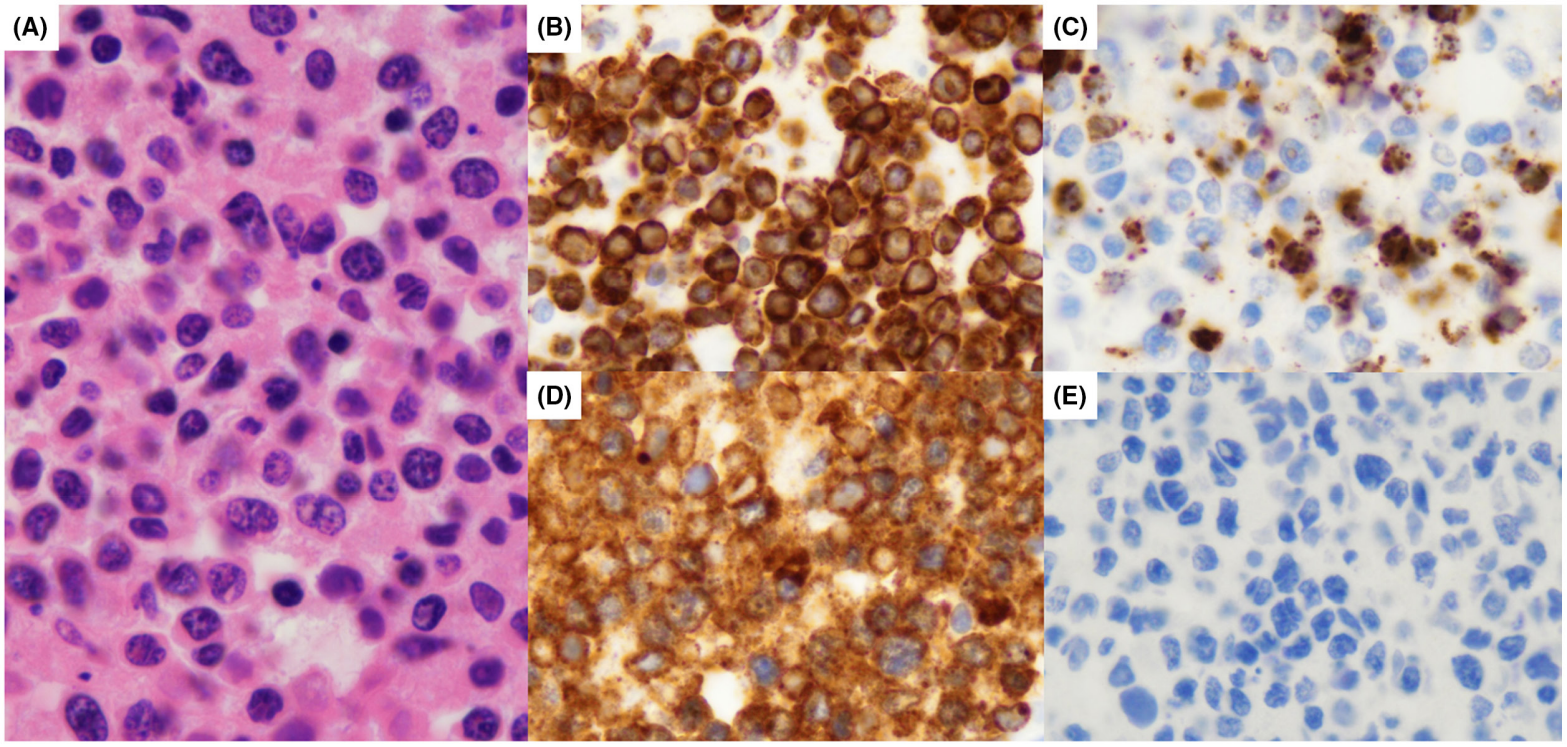
(A) Histologic sections of the bone marrow core biopsy showing diffuse involvement by a proliferation of atypical mononuclear cells and abundant coagulative necrosis. The atypical cells are intermediate to large in size and have a moderate amount of eosinophilic cytoplasm, angulated to irregularly shaped nuclei, hyperchromatic to vesicular chromatin, and prominent nucleoli. Immunohistochemical stains show that the population of interest expresses CD45, CD2, CD3 (B), perforin, and granzyme B (C) and does not express CD4, CD5, CD7, CD8, and TIA‐1; this immunophenotype suggests an atypical T‐cell population. These cells are positive for CD30 (D) and are negative for ALK1 (E). The morphologic and immunophenotypic findings are most consistent with a CD30+ T‐cell lymphoma, with an ALK negative anaplastic large cell lymphoma favored, though subsequent fluorescence in‐situ hybridization studies showed an intact IRF4/ DUSP22 gene. Further cytogenetic studies were unable to performed due to an absence of interphase and metaphase cells. Material was not available for flow cytometry. (Images at 1000x)

A bone marrow biopsy was then obtained revealing cluster differentiation (CD) 30 positive T‐cell lymphoma without evidence of significant hemophagocytosis. Extensive necrosis was also observed within the bone marrow (see Figure 1); fluorescent in situ hybridization was unable to be completed; however, anaplastic lymphoma kinase (ALK) negative anaplastic large cell lymphoma was favored.

## 4 OUTCOME AND FOLLOW‐UP

The patient's newly diagnosed T‐cell lymphoma was favored to be the likely driver of a number of her acute medical problems, including the encephalopathy, liver dysfunction, and worsening anemia via infiltration of the bone marrow. The patient was offered chemotherapy and corticosteroids for improvement in her symptoms and quality of life; however, her family declined treatment and chose to pursue hospice. A repeat ferritin obtained eleven days after the initial value and prior to discharge from the hospital remained elevated at >40,000 ng/ml. The patient was discharged under hospice care and passed away shortly thereafter.

## 5 DISCUSSION

Here, we have described a case of a patient presenting with markedly elevated ferritin levels who was ultimately diagnosed with T‐cell lymphoma, presumed anaplastic large cell lymphoma (ALCL) subtype, without a diagnosis of associated HLH. Peripheral T‐cell lymphomas (PTCL) are relatively rare, comprising less than 15% of non‐Hodgkin lymphoma diagnoses with ALCL as the second most common type (2%) behind peripheral T‐cell lymphoma, not otherwise specified (NOS).^21^ Interestingly, ALCL is the more common subtype to involve the central nervous system, which likely in‐part explains our patient's significant encephalopathy despite her unremarkable lumbar puncture. While hematologic malignancy is often noted as being a major cause of marked ferritin elevations in adults,^8^, ^9^ most cases are found in concert with HLH and it is unclear how specific an elevated ferritin alone is toward the diagnosis of T‐cell lymphoma. As reported, our patient did not meet criteria for HLH as defined in the HLH‐2004 study.^5^ However, given the strong connection between hematologic malignancy, elevated ferritin, and HLH, we could hypothesize that an elevated ferritin may be an early signal of HLH in patients with T‐cell lymphoma. Extreme hyperferritinemia and HLH may also exist on a spectrum, with secondary HLH likely to develop whether the underlying condition is left untreated. Additionally, there may be a distinct ferritin level at which the specificity for hematologic malignancy is greater, given these cases often show the widest range and highest values among non‐HLH associated causes of hyperferritinemia in adults.^8^, ^9^ Unfortunately, follow‐up with this patient was limited due to the advanced nature of her condition and decision to pursue hospice care.

Most reported cases of PTCL associated with HLH and hyperferritinemia in the literature involve extranodal nature killer/T‐cell lymphoma, nasal subtype with very few investigating the association of marked hyperferritinemia, HLH, and ALCL.^14^, ^15^, ^17^ However, of those reported cases of ALCL with associated hyperferritinemia and/or HLH, poorer outcomes and decreased overall survival have been demonstrated.^19^, ^20^ This observation highlights the importance of arriving at a timely and accurate diagnosis in cases of hyperferritinemia resulting from T‐cell lymphoma (with or without HLH), as diagnostic delay may impact progression of disease and prognosis.

In conclusion, in the adult population, high ferritin levels are less likely to signify rare conditions such as HLH and alternative investigation should be initiated early in the clinical course to identify more common etiologies, including hematologic malignancy.^8^, ^9^, ^11^ Our case demonstrates a unique case of PTCL presenting with marked ferritin elevation without associated HLH, potentially suggestive that HLH exists on a spectrum of disease.

## AUTHOR CONTRIBUTIONS

GL participated in the clinical care of this patient and wrote the manuscript under the mentorship of BW. SD participated in the clinical care of this patient and wrote the manuscript under the mentorship of BW. LF participated in the clinical care of this patient, provided pathology images, and contributed to the manuscript. BW supervised case report formulation, aided in the diagnosis of disease, and was the physician of record.

## CONFLICT OF INTEREST

All authors declare no conflicts of interest.

## ETHICAL APPROVAL

All procedures followed were in accordance with the ethical standards of the responsible committee on human experimentation and with the Helsinki Declaration of 1975, as revised in 2000. This study did not require IRB approval and was not part of a registered clinical trial.

## CONSENT

Written informed consent was obtained from the patient's family prior to manuscript composition.

## Data Availability

The data that support the findings of this study are available from the corresponding author upon reasonable request.
